# Cervical cancer screening coverage and its related knowledge in southern Malawi

**DOI:** 10.1186/s12889-022-12547-9

**Published:** 2022-02-14

**Authors:** Sibylle Gerstl, Lawrence Lee, Robin C. Nesbitt, Christopher Mambula, Hartini Sugianto, Twambilire Phiri, James Kachingwe, Augusto Eduardo Llosa

**Affiliations:** 1 Epicentre / Médecins Sans Frontières (MSF), Paris, France; 2 Médecins Sans Frontières (MSF), Paris, France; 3 Médecins Sans Frontières (MSF), Blantyre, Malawi; 4 Ministry of Health, Blantyre, Malawi

**Keywords:** Cervical cancer, Cervical cancer screening, Precancerous lesions, Malawi, Coverage survey, Cross sectional study, Knowledge and attitude, Health behaviours, Barriers, Reproductive health

## Abstract

**Background:**

Cervical cancer (CC) is the fourth most common cancer among women worldwide and Malawi has the world’s highest rate of cervical cancer related mortality. Since 2016 the National CC Control Strategy has set a screening coverage target at 80% of 25-49-year-old women. The Ministry of Health and Médecins Sans Frontières (MSF) set up a CC program in Blantyre City, as a model for urban areas, and Chiradzulu District, as a model for rural areas. This population-based survey aimed to estimate CC screening coverage and to understand why women were or were not screened.

**Methods:**

A population-based survey was conducted in 2019. All resident consenting eligible women aged 25-49 years were interviewed (*n* = 1850) at households selected by two-stage cluster sampling. Screening and treatment coverage and facilitators and barriers to screening were calculated stratified by age, weighted for survey design. Chi square and design-based F tests were used to assess relationship between participant characteristics and screening status.

**Results:**

The percentage of women ever screened for CC was highest in Blantyre at 40.2% (95% CI 35.1-45.5), 38.9% (95% CI 32.8-45.4) in Chiradzulu with supported CC screening services, and lowest in Chiradzulu without supported CC screening services at 25.4% (95% CI 19.9-31.8). Among 623 women screened, 49.9% (95% CI 44.0-55.7) reported that recommendation in the health facility was the main reason they were screened and 98.5% (95% CI 96.3-99.4) recommended CC screening to others. Among 1227 women not screened, main barriers were lack of time (26.0%, 95% CI 21.9-30.6), and lack of motivation (18.3%, 95% CI 14.1-23.3). Overall, 95.6% (95% CI 93.6-97.0) of women reported that they had some knowledge about CC. Knowledge of CC symptoms was low at 34.4% (95% CI 31.0-37.9) and 55.1% (95% CI 51.0-59.1) of participants believed themselves to be at risk of CC.

**Conclusion:**

Most of the survey population had heard about CC. Despite this knowledge, fewer than half of eligible women had been screened for CC. Reasons given for not attending screening can be addressed by programs. To significantly reduce mortality due to CC in Malawi requires a comprehensive health strategy that focuses on prevention, screening and treatment.

## Background

Cervical cancer is the fourth most common cancer among women worldwide for both incidence and mortality [1, 2], and approximately 90% of cervical cancer deaths occur in low- and middle-income countries [3]. Cervical cancer is particularly devastating to individuals, communities and countries as it is a painful cancer that affects women during childbearing and economically active years [4–6].

Cervical cancer is largely a preventable disease; primary prevention and control strategies including human papillomavirus (HPV) vaccination and early detection and treatment of precancerous lesions have contributed to the reduction in disease and mortality burden in many high-income settings with strong health and social systems [2, 7–9]. Cervical cancer mortality has thus become one indicator for functioning and equity of a health care system and has been considered a “a disease of the poor” [3].

Malawi has the highest mortality related to cervical cancer, with 51.5 deaths/100,000/year. This is twice the rate in Eastern Africa (28.6/100,000/year) and seven times the global rate (7.3/100,000/year) [10]. Only surpassed by Swaziland, Malawi has the second highest cervical cancer age-standardized incidence rate in the world (67.9/100,000/year). This compares to a global cervical cancer age-standardized incidence rate of 13.3/100,000/year, and to that of Eastern Africa, the region with the highest cervical cancer incidence rates, with 40.1.7/100,000/year [6, 10, 11].

The high prevalence of Human Immunodeficiency Virus (HIV) among women 15-49 years overall [12, 13] in addition to HPV infection, harboured by around 4.8% of the women in Malawi [14–19], puts Malawian women at higher risk of cervical cancer [20–22].

Health services in Malawi are provided by public, private for profit and private not for profit sectors. Its health system is organized at four levels: community, primary, secondary and tertiary. These different levels are linked to each other through an established referral system. Malawi’s health care services, however, experience shortages of essential medical products and technologies and health care financing remains a challenge [23]. In 2016, the Malawi Ministry of Health (MoH) developed the National Cervical Cancer Control Strategy outlining comprehensive interventions to mitigate the burden of cervical cancer [24]. Within this ‘Cervical Cancer Control Programme‘ the country adopted the single visit “screen and treat” approach, using visual inspection with acetic acid (VIA) followed by cryotherapy or thermo-coagulation at primary health facility level and with several referral hospitals offering diagnostic and curative cancer services. Cervical cancer screening is mentioned in the ‘Essential Health Plan’, and public health facilities provide services for free. There is, however, a need for scaling-up VIA screening services in health facilities and increasing resources, such as better infrastructure and human resources, for cervical cancer screening [6]*.* Current recommendations for VIA screening in Malawi are that women 25-49 years are screened once every 3 - 5 years, with yearly screening among HIV positive women. A national target of 80% screening coverage has been set for women aged 25-49 years being screened with VIA for the first time within the last 12-months [24]. Cervical cancer screening coverage increased from 9% in 2011 to 26.5% in 2015 nationwide [25]. According to the Health Sector Joint Annual Review Meeting held by the Directorate of Reproductive Health Services in the MoH on 21 September 2021, only 34% of eligible women were screened for cervical cancer between July 2020 and June 2021. Today cervical cancer screening coverage remains well below the target.

Studies on knowledge and awareness of cancer cervical screening carried out in Malawi and surrounding countries showed a consistent mix of screening barriers: little knowledge on symptoms or signs of the disease, lack of information of the screening programme and limited access to screening and treatment [18, 26–34].

Médecins Sans Frontières France (MSF) has been operational in Malawi since 1986 and involved in HIV care since 1997. In 2018, in partnership with the MoH, MSF set up a comprehensive cervical cancer program in Blantyre City, as a model for urban areas, and Chiradzulu District, as a model for rural areas, both situated in the South of the country. By following the “screen and treat” approach the cancer screening component includes health-facility based information sessions and VIA. Immediately after testing, VIA positive patients are offered treatment using thermal ablation in the same health facility by qualified health personnel in Blantyre City and Chiradzulu District. Precancer lesions not treatable by thermal ablation and suspected cancer are referred to a higher level care facility for further diagnosis and treatment. All screen and treat steps are backed up with quality controls. This population-based survey aimed to estimate cervical cancer screening coverage in a representative sample of eligible women in Blantyre City and Chiradzulu District, and to understand why women were or were not screened in order to support the MoH improve cervical cancer screening uptake in the different areas.

## Methods

### Survey design and survey population

In 2019, a cross-sectional population-based survey was conducted to estimate the prevalence of cervical cancer screening, and to collect reasons for screening among women 25 - 49 years old residing in Blantyre City and Chiradzulu District at the time of the survey. The target sample size was 1815 women in Blantyre City and Chiradzulu District combined.

### Survey area and sampling procedure

The survey was carried out in three areas, or strata, one in Blantyre City and two in Chiradzulu District. The survey utilized geospatial simple random sampling in Blantyre City, where one GPS coordinate identified one household, all resident consenting eligible women were interviewed. In Chiradzulu District, two-stage cluster sampling was utilized. First, Chiradzulu District was divided into two strata according to proximity to a cervical cancer screening facility supported by MSF; the stratification of the enumeration areas was done by the MSF survey team with the help of the National Statistical Office in Zomba and the health authorities at all levels in Chiradzulu District. Of the 330 EAs (total of 1243 villages) in Chiradzulu District, 161 were classified as having access to MSF-supported cervical cancer services and 169 were classified as without access to MSF-supported cervical cancer services. Within each strata 30 enumeration areas were selected as clusters in the first stage using probability proportional to size. For each cluster, one village was selected in the second stage using systematic random sampling, with 25 households systematically selected and all resident consenting eligible women interviewed.

For simplicity, the names of the three strata will be abbreviated as follows: (1) Blantyre City strata as ‘Blantyre’, (2) Chiradzulu District with access to MSF-supported cervical cancer services as ‘Chiradzulu with supported CC screening services’ and, (3) Chiradzulu District without access to MSF-supported cervical cancer services as ‘Chiradzulu without CC supported screening services’.

### Data collection and questionnaire

The questionnaire was developed in English and translated into Chichewa, the local language in southern Malawi spoken by the majority of the population. It included questions on knowledge and awareness of cervical cancer, risk factors and prevention, whether the women had ever received cervical cancer screening, and if so details about the screening (including date, location, reasons) and treatment if they received any. Cervical cancer screening was confirmed both by oral history of the interviewee (self-reported) and by the presence of a health passport that contained this information (verified by health passport). The questionnaire was context-adapted based on two models to measure knowledge and awareness of cervical cancer, the ‘Cervical Cancer Awareness Measure Toolkit’ and the ‘Health Belief Model Scale for Cervical Cancer’ [35, 36]. It was further tested during the training of the survey team and in a 1-day pilot survey. Data were collected using KoBo Collect (https://kobo.msf.org) on electronic tablets.

### Data analysis

Data were analysed using Stata version 14.1 (Stata Corp, Texas, USA). Screening and treatment coverage were calculated stratified by age, weighted for survey design. Proportion of women reporting facilitators and barriers to screening were also reported. Chi square and design-based F tests were used to assess relationship between participant characteristics and screening status in the different geographical areas. Interviews were weighted based on the inverse of probability of selection separately by stratum. In the pooled analysis artificial clusters using the administrative boundaries of the wards were introduced in Blantyre due to the different sampling methodology between the three strata.

## Results

### Survey profile

The survey took place from 24 September to 26 October 2019. A total of 3642 households were visited, and 1824 out of 1885 (96.8%) households with at least one eligible woman aged 25-49 years were interviewed, 45 (2.4%) refused to participate in the survey. The survey population included a total of 1850 women: 343 (18.5%) in Blantyre, 755 (40.8%) in Chiradzulu with supported CC screening services and 752 (40.6%) in Chiradzulu without supported CC screening services.

### Sociodemographic characteristics of survey participants

The mean age of participants was 34 years overall (mean = 33.9, std. error = 0.2659, 95% CI: 33.3 - 34.4) and did not differ by survey strata, *p* = 0.13 (Table 1). Literacy was higher in Blantyre where 84.8% of participants were literate compared to 76.3% in Chiradzulu without supported CC screening services and 69.4% in Chiradzulu with supported CC screening services, *p* < 0.001. Working outside of the home was more common in Blantyre than in either strata in Chiradzulu, and the proportion of women reporting the highest category of average monthly household income was higher in Blantyre at 43.7% compared to 12.2 and 8.0% in Chiradzulu with and without supported CC screening services respectively, p < 0.001. The proportion of HIV positive participants on anti-retroviral therapy (ART) was lower in Blantyre at 14.9% than in Chiradzulu with supported CC screening services at 25.6% and without supported CC screening services at 22.6%, *p* = 0.006.

**Table 1:** Sociodemographic characteristics of survey participants (*n* = 1850), Blantyre City and Chiradzulu District

	All strata pooled				Blantyre City			Chiradzulu District with supported cervical cancer screening services			Chiradzulu District without supported cervical cancer screening services		
	*n* = 1.850	Col %	95% CI	*p*-value	*n* = 343	Col %	95% CI	*n* = 755	Col %	95% CI	*n* = 752	Col %	95% CI
Average age (years)	34.0				33.7		33.0-34.4	34.2		33.7-34.6	34.3		33.7-34.8
Age groups (years)													
25-35	1125	63.3	59.6-66.8	0.130	221	64.4	59.2-69.3	460	60.9	58.0-63.7	444	59.0	54.8-63.2
36-49	725	36.7	33.2-40.4		122	35.6	30.7-40.8	295	39.1	36.3-42.0	308	41.0	36.8-45.2
Marital status													
Married / living together	1437	75.8	71.2-79.8	p<0.001	257	74.9	70.0-79.3	599	79.3	76.4-82.0	581	77.3	73.1-81.0
Separated / divorced	286	13.9	11.4-16.8		45	13.1	9.9-17.1	117	15.5	13.2-18.1	124	16.5	13.5-20.0
Widow	87	4.5	2.9-6.9		15	4.4	2.6-7.1	33	4.4	3.2-6.0	39	5.2	3.7-7.1
Single	40	5.8	3.5-9.7		26	7.6	5.2-10.9	6	0.8	0.3-2.2	8	1.0	0.4-2.5
Literacy													
Illiterate	461	18.2	15.2-21.7	p<0.001	52	15.2	11.7-19.4	231	30.6	26.0-35.6	178	23.7	19.0-29.1
Literate	1389	81.8	78.3-84.8		291	84.8	80.6-88.3	524	69.4	64.4-74.0	574	76.3	70.9-81.0
Occupation													
Household work	874	41.7	36.3-47.2		134	39.1	34.0-44.4	384	50.9	40.3-61.3	356	47.3	38.9-56.0
Farming	444	10.1	7.9-12.8		12	3.5	2.0-6.1	217	28.7	19.7-39.9	215	28.6	20.9-37.8
Employed (for wages)	101	15.4	11.1-21.0		69	20.1	16.2-24.7	18	2.4	1.0-5.4	14	1.9	0.9-3.6
Business (self-employed)	333	27.4	23.3-31.9		109	31.8	27.0-36.9	100	13.2	9.3-18.4	124	16.5	13.2-20.4
Casual labour	91	5.2	3.6-7.2		18	5.2	3.3-8.2	33	4.4	2.3-8.2	40	5.3	2.9-9.7
Other occupation	7	0.2	0.1-1.3		1	0.3	0.0-2.1	3	0.4	0.1-1.2	3	0.4	0.1-1.2
Average monthly household income (MK - Malawian Kwacha)^a^													
< 20,000 MK	1097	35.4	31.6-39.4	p<0.001﻿	82	23.9	19.7-28.7	463	61.3	52.0-69.9	552	73.4	67.2-78.8
20,000-40,000 MK	451	29.7	25.1-34.7		111	32.4	27.6-37.5	200	26.5	20.9-32.9	140	18.6	14.3-23.9
> 40,000 MK	302	34.9	30.1-40.0		150	43.7	38.5-49.1	92	12.2	8.3-17.6	60	8.0	5.7-11.0
HIV Status													
Negative	1402	80.6	76.9-83.7	0.006	284	82.8	78.4-86.5	554	73.4	69.8-76.7	564	75.0	71.2-78.4
Positive (on anti-retroviral therapy, ART)	414	17.3	14.0-21.1		51	14.9	11.5-19.1	193	25.6	22.3-29.1	170	22.6	19.1-26.5
Unknown or no answer given	34	2.1	1.2-4.1		8	2.3	1.2-4.6	8	1.0	0.4-2.5	18	2.4	1.3-4.3

^a^ MK = Malawian Kwacha. 1 MK = 0.0012 Euros as of 25/11/2019 (20,000 MK = 24.54 Euros; 40,000 MK = 49.08 Euros)

### Cervical cancer screening coverage

**Fig. 1:**
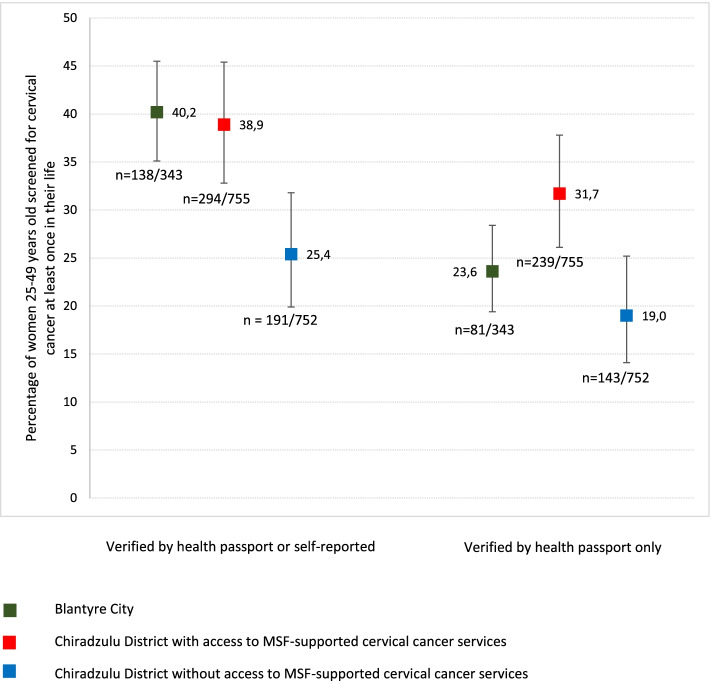
Estimated percentage of women 25-49 years who have ever been screened for cervical cancer by stratum, Blantyre City and Chiradzulu District, Southern Malawi, 2019

The percentage of women aged 25-49 years ever screened for cervical cancer (either verified by health passport or self-reported) differed by stratum, and was highest in Blantyre at 40.2% (138/343, 95% CI 35.1-45.5), 38.9% (294/755, 95% CI 32.8-45.4) in Chiradzulu with supported CC screening services, and lowest in Chiradzulu without supported CC screening services at 25.4% (191/752, 95% CI 19.9-31.8) (Fig. 1). Screening coverage of women aged 25-49 years was statistically significantly greater in Chiradzulu with supported CC screening services compared to Chiradzulu without supported CC screening services (*p* = 0.003). The estimated screening coverage considering only those verified in the health passport, was 23.6% (81/343, 95% CI 19.4-28.4) in Blantyre; 31.7% (239/755, 95% CI 26.1-37.8) in Chiradzulu with access to supported CC services, and 19.0% (143/752, 95% CI 14.1-25.2) in Chiradzulu without access to supported CC services.

The percentage of survey participants screened multiple times for cervical cancer was 9.9% (34/343, 95% CI 7.2-13.6) in Blantyre; 6.9% (52/755, 95% CI 4.7-10.0) in Chiradzulu with access to supported CC screening services; and 3.2% (24/752, 95% CI 2.1-4.8) in Chiradzulu without access to supported CC screening services.

### Results of cervical cancer screening

Among the survey participants who were screened, 4 (0.2, 95% CI 0.1-0.6) were VIA positive (3 verified in health passport and 1 self-reported) and 1 (0.1, 95% CI 0.01-0.4) was identified as suspect cancer, verified in the health passport.

Three of the four VIA positive survey participants and the suspect cancer case received treatment. The VIA positive participant that did not receive treatment reported that treatment was not necessary. Three out of the four VIA positive survey participants and the suspect cancer case were HIV positive on ART.

### Characteristics associated with cervical cancer screening among women screened in the different geographical areas

Considering characteristics associated with screening by survey site, older age and literacy were consistently associated with being screened for cervical cancer in all three geographical areas, whereas other characteristics differed. Education was associated with screening in Blantyre only, and income level was associated with screening in Blantyre and Chiradzulu without access to supported CC screening services, but not Chiradzulu with access to supported CC screening services. HIV status significantly was associated with screening in both Chiradzulu sites. In Blantyre, where overall proportion of HIV positive participants was lower, there was no association between screening and HIV status (Table 2).

**Table 2:** Characteristics associated with cervical cancer screening in survey participants, Blantyre City and Chiradzulu District, Malawi

	All strata pooled							Blantyre City						
	Never screened			Screened				Never screened			Screened			
	n	Row %	95% CI	n	Row %	95% CI	p-value	n	Row %	95% CI	n	Row %	95% CI	p-value
Age groups (Years)														
25-35	782	66.6	62.7-70.3	343	33.4	29.7-37.3	p<0.001﻿	143	64.7	58.1-70.8	78	35.3	29.2-41.9	0.013
36-49	445	55.2	49.3-61.1	280	44.8	38.9-50.7		62	50.8	42.0-59.6	60	49.2	40.4-58.0	
*Total*	*1227*	*62.4*	*58.8-65.8*	*623*	*37.6*	*34.2-41.2*		*205*	*59.8*	*54.5-64.9*	*138*	*40.2*	*35.1-45.5*	
Literacy														
Illiterate	341	73.8	67.4-79.2	120	26.2	20.8-32.6	p<0.001﻿	38	73.1	59.5-83.4	14	26.9	16.6-40.5	0.035
Literate	886	59.8	55.8-63.6	503	40.2	36.4-44.2		167	57.4	51.6-63.0	124	42.6	37.0-48.4	
*Total*	*1227*	*62.4*	*58.8-65.8*	*623*	*37.6*	*34.2-41.2*		*205*	*59.8*	*54.5-64.9*	*138*	*40.2*	*35.1-45.5*	
Education Level														
Less than primary school	782	69.2	63.2-74.5	345	30.8	25.5-36.8	p<0.001﻿	98	68.5	60.4-75.6	45	31.5	24.4-39.6	0.010
Primary and less than secondary	326	61.0	54.4-67.2	182	39.0	32.8-45.6		59	59.0	49.1-68.2	41	41.0	31.8-50.9	
Secondary or higher school	114	49.7	42.3-57.1	95	50.3	42.9-57.7		48	48.5	38.8-58.3	51	51.5	41.7-61.2	
Missing data	5	34.6	5.3-83.3	1	65.4	16.7-94.7		0	0.0		1	100.0		
*Total*	*1227*	*62.4*	*58.8-65.8*	*623*	*37.6*	*34.2-41.2*		*205*	*59.8*	*54.5-64.9*	*138*	*40.2*	*35.1-45.5*	
Average monthly household income (MK - Malawian Kwacha)^a^														
< 20,000 MK	767	68.6	62.7-74.0	330	31.4	26.0-37.3	0.001	54	65.9	54.9-75.3	28	34.1	24.7-45.1	0.010
20,000-40,000 MK	292	66.8	61.4-71.8	159	33.2	28.2-38.6		75	67.6	58.3-75.6	36	32.4	24.4-41.7	
> 40,000 MK	168	51.6	43.7-59.3	134	48.4	40.7-56.3		76	50.7	42.7-58.6	74	49.3	41.4-57.3	
*Total*	*1227*	*62.4*	*58.8-65.8*	*623*	*37.6*	*34.2-41.2*		*205*	*59.8*	*54.5-64.9*	*138*	*40.2*	*35.1-45.5*	
HIV Status														
Negative	963	63.6	60.1-67.0	439	36.4	33.0-39.9	0.363	172	60.6	54.7-66.1	112	39.4	33.9-45.3	0.752
Positive (on anti-retroviral therapy, ART)	237	57.3	50.2-64.2	177	42.7	35.8-49.8		29	56.9	43.0-69.7	22	43.1	30.3-57.0	
Unknown or no answer given	27	59.8	30.8-83.2	7	40.2	16.8-69.2		4	50.0	19.9-80.1	4	5.0	19.9-80.1	
*Total*	*1227*	*62.4*	*58.8-65.8*	*623*	*37.6*	*34.2-41.2*		*205*	*59.8*	*54.5-64.9*	*138*	*40.2*	*35.1-45.5*	

	Chiradzulu District with supported cervical cancer screening services							Chiradzulu District without supported cervical cancer screening services						
	Never screened			Screened				Never screened			Screened			
	n	Row %	95% CI	n	Row %	95% CI	p-value	n	Row %	95% CI	n	Row %	95% CI	p-value
Age groups (Years)														
25-35	295	64.1	56.5-71.1	165	35.9	28.9-43.5	0.022	344	77.5	70.5-83.2	100	22.5	16.8-29.5	0.035
36-49	166	56.3	49.2-63.1	129	43.7	36.9-50.8		217	70.5	62.3-77.5	91	29.5	22.5-37.7	
*Total*	*461*	*61.1*	*54.5-67.3*	*294*	*38.9*	*32.7-45.5*		*561*	*74.6*	*68.1-80.2*	*191*	*25.4*	*19.8-31.9*	
Literacy														
Illiterate	156	67.5	60.0-74.3	75	32.5	25.7-40.0	0.028	147	82.6	72.4-89.5	31	17.4	10.5-27.6	0.025
Literate	305	58.2	50.7-65.3	219	41.8	34.7-49.3		414	72.1	65.6-77.8	160	27.9	22.2-34.4	
*Total*	*461*	*61.1*	*54.5-67.3*	*294*	*38.9*	*32.7-45.5*		*561*	*74.6*	*68.1-80.2*	*191*	*25.4*	*19.8-31.9*	
Education Level														
Less than primary school	320	62.4	55.2-69.1	193	37.6	30.9-44.8	0.351	364	77.3	69.7-83.4	107	22.7	16.6-30.3	0.125
Primary and less than secondary	110	59.8	50.9-68.1	74	40.2	31.9-49.1		157	70.1	62.3-76.9	67	29.9	23.1-37.7	
Secondary or higher school	30	52.6	41.5-63.5	27	47.4	36.5-58.5		36	67.9	53.6-79.5	17	32.1	20.5-46.4	
Missing data	1	100.0		0	0.0			4	100.0		0	0.0		
*Total*	*461*	*61.1*	*54.5-67.3*	*294*	*38.9*	*32.7-45.5*		*561*	*74.6*	*68.1-80.2*	*191*	*25.4*	*19.8-31.9*	
Average monthly household income (MK - Malawian Kwacha)^a^														
< 20,000 MK	286	61.8	53.8-69.2	177	38.2	30.8-46.2	0.777	427	77.4	70.7-82.8	125	22.6	17.2-29.3	0.006
20,000-40,000 MK	118	59.0	50.4-67.1	82	41.0	32.9-49.6		99	70.7	59.9-79.6	41	29.3	20.4-40.1	
> 40,000 MK	57	62.0	51.8-71.1	35	38.0	28.9-48.2		35	58.3	45.1-70.5	25	41.7	29.5-54.9	
*Total*	*461*	*61.1*	*54.5-67.3*	*294*	*38.9*	*32.7-45.5*		*561*	*74.6*	*68.1-80.2*	*191*	*25.4*	*19.8-31.9*	
HIV Status														
Negative	360	65.0	57.9-71.5	194	35.0	28.5-42.1	p<0.001﻿	431	76.4	69.1-82.4	133	23.6	17.6-30.9	0.028
Positive (on anti-retroviral therapy, ART)	94	48.7	39.5-58.0	99	51.3	42.0-60.5		114	67.1	58.1-74.9	56	32.9	25.1-41.9	
Unknown or no answer given	7	87.5	57.9-97.3	1	12.5	2.7-42.1		16	88.9	62.4-97.5	2	11.1	2.5-37.6	
*Total*	*461*	*61.1*	*54.5-67.3*	*294*	*38.9*	*32.7-45.5*		*561*	*74.6*	*68.1-80.2*	*191*	*25.4*	*19.8-31.9*	

^a^ MK = Malawian Kwacha. 1 MK = 0.0012 Euros as of 25/11/2019 (20,000 MK = 24.54 Euros; 40,000 MK = 49.08 Euros)

### Reasons for cervical cancer screening

Among 623 women screened overall, 334 (49.9, 95% CI 44.0-55.7) reported that recommendation in the health facility was the main reason they were screened. This proportion was 48.6% (95% CI 40.2-57.0) in Blantyre, 60.5% (95% CI 52.0-68.5) in Chiradzulu with supported CC screening services, and 46.6% (95% CI 35.4-58.2) in Chiradzulu without supported CC screening services. Other common reasons included recommended by screening campaign (12.4% overall, 95% CI 9.0-16.7), self-volition (10.8, 95% CI 6.5-17.5) and recommended by family and friends (10.2, 95% CI7.1-14.4). Overall, 98.5% (95% CI 96.3-99.4) of women who were screened reported that they would recommend cervical cancer screening to others; this was consistent across all three strata (Table 3).

**Table 3:** Reasons for cervical cancer screening among women screened (*n* = 623), Blantyre City and Chiradzulu District, Malawi

	All strata pooled			Blantyre City			Chiradzulu District with supported cervical cancer screening services			Chiradzulu District without supported cervical cancer screening services		
	n	Col. %	95% CI	n	Col. %	95% CI	n	Col. %	95% CI	n	Col. %	95% CI
Main reason for cervical cancer screening												
Recommended in health facility	334	49.9	44.0-55.7	67	48.6	40.2-57.0	178	60.5	52.0-68.5	89	46.6	35.4-58.2
Self-volition	77	10.8	6.5-17.5	14	10.1	6.1-16.5	29	9.9	5.9-16.0	34	17.8	11.8-26.0
Recommended by family / friends	64	10.2	7.1-14.4	14	10.1	6.1-16.5	31	10.5	7.3-15.0	19	9.9	6.1-15.9
Recommended by screening campaigns	54	12.4	9.0-16.7	19	13.8	8.9-20.7	17	5.8	3.6-9.1	18	9.4	6.3-13.9
Recommended by media	37	8.4	5.6-12.5	13	9.4	5.5-15.6	17	5.8	3.4-9.8	7	3.7	1.7-7.8
Other reason	54	8.2	4.5-14.4	11	8.0	4.4-13.9	19	6.5	3.7-11.0	24	12.6	7.9-19.5
No reason given	3	0.1	0.0-0.4	0	0		3	1.0	0.3-3.2	0	0	
*Total*	*623*	*1*		*138*	*100*		*294*	*100*		*191*	*100*	
Would recommend cervical cancer screening to others												
No	10	1.5	0.6-3.7	2	1.4	0.4-5.7	8	2.7	1.2-6.3	0	0	
Yes	613	98.5	96.3-99.4	136	98.6	94.3-99.6	286	97.3	93.7-98.8	191	100	
*Total*	*623*	*100*		*138*	*100*		*294*	*100*		*191*	*100*	

### Reasons for not screening

Among 1227 women not screened overall, the main two reasons for not being screened were lack of time for screening (26.0, 95% CI 21.9-30.6), and lack of motivation to go for screening (18.3, 95% CI 14.1-23.3). Reasons for not being screened differed according to strata. In Blantyre, 30.2% (95% CI 24.3-36.9) of women reported lack of time for screening as the main reasons they were not screened and 20.1% (95% CI 15.0-26.1) reported lack of motivation. In Chiradzulu without supported CC screening services the most common reasons were that the location was not convenient (21.9, 95% CI 15.6-29.9), followed by lack of information (18.5, 95% CI 14.6-23.3), whereas lack of time for screening was less important, reported by 13.4% (95% CI 9.0-19.3). In Chiradzulu with supported CC screening services, lack of information 19.1% (95% CI 13.2-26.8), lack of time for screening (18.9, 95% CI 14.2-24.7), and lack of motivation 18.4% (95% CI 13.3-25.0) were the top reasons reported by similar proportions of women (Table 4).

**Table 4:** Reasons for not screening among women not screened (*n* = 1227), Blantyre City and Chiradzulu District, Malawi

	All strata pooled			Blantyre City			Chiradzulu District with supported cervical cancer screening services			Chiradzulu District without supported cervical cancer screening services		
	n	Col. %	95% CI	n	Col. %	95% CI	n	Col. %	95% CI	n	Col. %	95% CI
Main reason not screened												
Lack of time for screening	224	26.0	21.9-30.6	62	30.2	24.3-36.9	87	18.9	14.2-24.7	75	13.4	9.0-19.3
Lack of information about screening	216	13.7	10.3-18.1	24	11.7	7.9-16.9	88	19.1	13.2-26.8	104	18.5	14.6-23.3
Screening location not convenient	197	10.4	8.0-13.4	15	7.3	4.4-11.8	59	12.8	8.4-19.0	123	21.9	15.6-29.9
Lack of motivation to go for screening	187	18.3	14.1-23.3	41	20.1	15.0-26.1	85	18.4	13.3-25.0	61	10.9	7.2-16.0
Fear of screening	149	13.7	11.1-16.9	30	14.6	10.4-20.2	61	13.2	10.2-16.9	58	10.3	7.4-14.3
Lack of screening capacity at health facility	58	3.9	2.5-6.0	7	3.4	1.6-7.0	21	4.6	2.6-7.9	30	5.3	2.8-9.9
Other reason	41	3.4	2.0-5.8	7	3.4	1.6-7.0	8	1.7	0.8-3.7	26	4.6	3.1-6.9
No reason given	155	10.5	5.9-17.9	19	9.3	6.0-14.1	52	11.3	7.8-16.0	84	15.0	9.5-22.8
*Total*	*1227*	*100*		*205*	*100*		*461*	*100*		*561*	*100*	
Would be screened by men												
No	49	4.9	3.0-7.8	11	5.3	3.0-9.5	17	3.7	2.1-6.5	21	3.7	2.4-5.7
Yes	1171	94.3	91.3-96.3	192	93.7	89.3-96.3	441	95.7	93.0-97.3	538	95.9	93.8-97.3
Do not know	7	0.8	0.2-2.8	2	1.0	0.2-3.9	3	0.6	0.2-2.0	2	0.4	0.1-1.4
*Total*	*1227*	*100*		*205*	*100*		*461*	*100*		*561*	*100*	
Would pay for screening												
No	548	37.8	33.1-42.7	70	34.1	27.9-41.0	217	47.1	41.4-52.8	261	46.5	40.1-53.1
Yes	679	62.2	57.3-66.9	135	65.9	59.0-72.1	244	52.9	47.2-58.6	300	53.5	46.9-59.9
*Total*	*1227*	*100*		*205*	*100*		*461*	*100*		*561*	*100*	

Fear of screening was not a predominant concern, overall 149 (13.7, 95% CI 11.1-16.9) women who were not screened reported fear as the main reason for not screening (Table 4). Furthermore, only 49 (4.9, 95% CI 3.0-7.8) of women not screened said they were not willing to be screened by a man. Overall 62.2% (95% CI 57.3-66.9) of women who were not screened showed a willingness to pay for this service. This number was lower in both strata in Chiradzulu district than Blantyre (Table 4).

### Knowledge, awareness and beliefs about cervical cancer and cervical cancer screening

Overall, 95.6% (95% CI 93.6-97.0) of women reported that they had knowledge about cervical cancer, this proportion was similar among participants who had not been screened for cervical cancer themselves at 94.0% (95% CI 91.4-95.8, *p* = 0.069). Fewer women reported knowledge of *screening* for cervical cancer, and this did significantly differ between women who were screened themselves and those who were not screened, 74.8% (95% CI 70.3-78.9, *p* < 0.001) overall and 64.6% (95% CI 58.1-70.5, p < 0.001) among women who were not screened themselves. Knowledge of cervical cancer symptoms was significantly low at overall 34.4% (95% CI 31.0-37.9, p < 0.001) and 24.6% (95% CI 21.8-27.6, p < 0.001) among women who had not been screened for cervical cancer themselves (Table 5).

**Table 5:** Knowledge, awareness and beliefs about cervical cancer and cervical cancer screening in the survey participants

	Screened by health centers with supported cervical cancer services (health passport or self-report)			Screened by health centers without supported cervical cancer services (health passport or self-report)			Never screened for cervical cancer			Total			
	n	Col. %	95% CI	n	Col. %	95% CI	n	Col. %	95% CI	n	Col. %	95% CI	*p*-value
Knowledge of cervical cancer													
No	3	2.1	0.4-11.7	3	1.7	0.5-6.0	111	6.0	4.2-8.6	117	4.4	3.0-6.4	0.069
Yes	298	97.9	88.3-99.6	319	98.3	94.0-99.5	1116	94.0	91.4-95.8	1733	95.6	93.6-97.0	
*Total*	*301*	*100*		*322*	*100*		*1227*	*100*		*1850*	*100*		
Knowledge of cervical cancer screening													
No	21	6.4	2.6-15.0	37	9.3	6.1-14.0	448	34.4	28.6-40.7	506	24.5	20.5-29.0	p<0.001﻿
Yes	279	93.5	84.9-97.3	284	90.6	86.0-93.8	765	64.6	58.1-70.5	1328	74.8	70.3-78.9	
Do not know	1	0.1	0.0-1.0	1	0.1	0.0-0.5	14	1.1	0.4-2.7	16	0.7	0.3-1.7	
*Total*	*301*	*100*		*322*	*100*		*1227*	*100*		*1850*	*100*		
Knowledge of cervical cancer symptoms													
No	151	50.0	41.5-58.5	156	49.6	41.3-58.0	898	75.4	72.4-78.2	1205	65.6	62.1-69.0	p<0.001﻿
Yes	150	50.0	41.5-58.5	166	50.4	42.0-58.7	329	24.6	21.8-27.6	645	34.4	31.0-37.9	
Total	301	100		322	100		1227	100		1850	100		
Awareness of being at risk for cervical cancer themselves													
No	118	43.6	33.3-54.5	82	28.8	22.0-36.8	217	23.4	19.7-27.4	417	27.2	23.3-31.4	0.006
Yes	167	50.8	40.0-61.5	199	54.1	45.8-62.2	809	56.3	51.6-60.9	1175	55.1	51.0-59.1	
Do not know	16	5.6	2.6-11.6	41	17.1	10.1-27.3	201	20.3	16.1-25.3	258	17.7	14.4-21.6	
*Total*	*301*	*100*		*322*	*100*		*1227*	*100*		*1850*	*100*		
Awareness of possibility to prevent cervical cancer													
No	61	15.3	11.0-20.9	52	11.0	7.5-15.9	292	20.6	16.5-25.5	405	17.5	14.5-20.9	0.001
Yes	212	75.7	68.2-81.8	242	79.6	74.6-83.8	722	61.9	55.7-67.7	1176	68.1	64.2-71.8	
Do not know	28	9.0	5.0-15.9	28	9.4	5.6-15.5	213	17.5	13.6-22.3	269	14.4	11.8-17.5	
*Total*	*301*	*100*		*322*	*100*		*1227*	*100*		*1850*	*100*		
Awareness of possibility to cure cervical cancer													
No	50	13.8	7.8-23.0	60	13.1	8.3-20.0	277	21.2	17.7-25.2	387	18.2	15.7-21.0	0.001
Yes	240	84.7	76.2-90.5	244	81.9	74.0-87.9	798	67.5	63.0-71.6	1282	73.3	70.1-76.2	
Do not know	11	1.5	0.8-3.1	18	5.0	2.2-10.7	152	11.3	9.2-13.9	181	8.5	7.1-10.3	
*Total*	*301*	*100*		*322*	*100*		*1227*	*100*		*1850*	*100*		
Knowledge on prevention methods, such as:													
- Medical check-up, VIA screening, HPV vaccination													
True	287	94.6	86.9-97.9	300	91.7	84.8-95.7	1119	92.4	88.9-94.9	1706	92.5	89.6-94.7	0.602
False	10	4.8	1.7-12.9	16	4.8	2.2-10.2	69	5.2	3.4-7.8	95	5.0	3.4-7.3	
Do not know	4	0.6	0.2-1.6	6	3.4	1.6-7.3	39	2.4	1.4-4.2	49	2.5	1.6-3.8	
*Total*	*301*	*100*		*322*	*100*		*1227*	*100*		*1850*	*100*		
- Being faithful to a sexual partner													
True	270	85.5	74.6-92.2	290	89.6	83.7-93.5	1033	87.5	84.1-90.2	1593	87.8	84.3-90.6	0.332
False	25	12.0	6.0-22.5	23	5.3	2.8-9.9	143	8.5	6.6-10.9	191	8.1	6.1-10.6	
Do not know	6	2.5	0.6-10.6	9	5.1	2.4-10.8	51	4.0	2.6-6.2	66	4.1	2.7-6.3	
*Total*	*301*	*100*		*322*	*100*		*1227*	*100*		*1850*	*100*		
- Delaying sexual debut													
True	173	59.9	51.3-67.9	206	63.6	57.4-69.3	663	55.7	51.2-60.1	1042	58.3	54.8-61.6	0.134
False	84	28.8	18.2-42.4	66	18.7	14.4-23.9	352	27.1	22.6-32.2	502	25.1	21.7-28.8	
Do not know	44	11.3	5.6-21.4	50	17.7	12.9-23.8	212	17.2	13.9-21.1	306	16.6	13.8-19.8	
*Total*	*301*	*100*		*322*	*100*		*1227*	*100*		*1850*	*100*		
Knowledge of being HIV positive as a risk factor													
No	50	18.9	12.1-28.3	36	11.5	7.1-18.0	192	15.6	12.1-19.9	278	14.9	12.1-18.2	0.222
Yes	221	68.5	56.0-78.8	255	79.7	72.4-85.4	875	70.0	66.0-73.6	1351	72.3	69.1-75.4	
Do not know	30	12.6	6.1-24.3	31	8.8	4.8-15.7	160	14.4	11.7-17.6	221	12.8	10.8-15.0	
*Total*	*301*	*100*		*322*	*100*		*1227*	*100*		*1850*	*100*		
Believe in witchcraft as cause for cervical cancer													
No	177	63.9	55.4-71.6	219	72.0	63.5-79.2	670	60.0	53.1-66.6	1066	63.6	58.6-68.4	0.03
Yes	82	25.1	18.6-33.1	62	13.9	9.2-20.5	360	26.1	22.0-30.7	504	22.8	19.4-26.5	
Do not know	42	11.0	6.3-18.6	41	14.1	9.1-21.2	197	13.9	10.6-17.9	280	13.6	10.9-16.8	
*Total*	*301*	*100*		*322*	*100*		*1227*	*100*		*1850*	*100*		

Overall, 55.1% (95% CI 51.0-59.1) of participants believed themselves to be at risk of cervical cancer, this was highest among those never screened themselves (56.3, 95% CI 51.6-60.9, *p* = 0.006). Additionally, 20.3% (95% CI 16.1-25.3) of the women not screened did not know if they were at risk of cervical cancer. In contrast, a high proportion of women reported awareness that cervical cancer can be prevented (68.1, 95% CI 64.2-71.8) and cured (73.3, 95% CI 70.1-76.2), and this was higher among women who were screened compared to women not screened, *p* = 0.001 for both indicators. There was a similar and relatively high level of knowledge on prevention methods (having regular medical check-ups, being faithful to their partner, delaying sexual debut), and HIV as a risk factor among women who were and were not screened. However, 36.4% of women believed in witchcraft as cause for cervical cancer or were not sure about it (Table 5).

## Discussion

Overall, despite relatively high knowledge of cervical cancer, cervical cancer screening coverage in Blantyre City and Chiradzulu District remained well below the national goal of 80%. Coverage was highest in Blantyre with almost every second eligible women screened, compared to slightly more than every third women screened in Chiradzulu with supported CC screening services and only every fourth women in Chiradzulu without supported CC screening services. Few women in our survey were screened more than once. This suggests that screening programs continue to reach women with cervical cancer screening for the first time, and that there remains a rather long way to go to reach routine cervical cancer screening among eligible women.

The few surveys that have been published on cervical cancer screening coverage in Malawi and other African countries were in areas with low coverage where no more than a quarter of the survey population had been screened, with the exception of Cameroon where almost half of the survey population had been screened [30, 37–41]. Our survey therefore provides an important contribution to this topic.

Knowledge of cervical cancer, risk factors, and possible prevention methods are present in the survey population. However, less than half of the women who have heard of cervical cancer screening have been screened. Likewise, only slightly more than one-third of women who said they believe they are at risk for cervical cancer have been screened. This suggests that knowledge of cervical cancer screening and awareness of being themselves at risk of cervical cancer are not the main barriers to screening in this population. Although the survey participants know that screening is important, and more than half thought they were personally at risk, it did not translate into action. Increasing knowledge about cervical cancer and screening alone is therefore not enough, practical actions that women can do to prevent cervical cancer should be clearly communicated. Information campaigns on cervical cancer risk and prevention possibilities must be reinforced, and specifically target women who have not yet accessed cervical cancer screening services.

These results are consistent with recent qualitative surveys, which showed high awareness but low uptake of cervical cancer screening not only in Malawi [34, 37, 42], and also in other African countries [27, 28, 38, 40, 43–45]. The results are also in line with surveys carried out in Kenya, where higher screening rates were observed in women with higher levels of education in the highest income quintile and living in urban areas [41, 46].

Overall, almost all women reported knowledge of cervical cancer, and among those not screened, very few reported fear as a deterrent to screening, or that they would not be comfortable being screened by a man. In Blantyre City, an urban setting with better access to care the main reason for not being screened was lack of time, in Chiradzulu District with access to free and enhanced CC screening services the main reason was lack of information and in Chiradzulu without access to free and enhanced CC screening services the main reason was inconvenient location. While the predominant reasons for not being screened differed by strata, and can be addressed specifically in each setting, they were consistent in that the barriers were practical, mutable constructs that are amenable to intervention [34, 47]. They included supply-side barriers, (such as lack of time, and lack of access) which can be addressed by the health system, for example by extending hours of service provision, and accessibility of cervical cancer screening sites. In addition, demand-side barriers were important (such as lack of motivation and lack of information), which can be addressed by adapting and scaling up information campaigns. These results are internally consistent with the reasons women reported for screening, which for a majority was due to a recommendation in a health facility, indicating that cervical cancer screening was often an ‘add-on’ service that women receive when they are already seeking care for other services. The higher proportion of women with HIV among women screened in Chiradzulu District is also consistent with the understanding that women undergo cervical cancer screening while they are already in the health facility for other services.

Among all interviewed women who underwent cervical cancer screening in the past, less than 1% were VIA positive. This is a surprisingly low VIA positivity rate in light of previous studies, including a country wide study reporting 10% VIA positivity or suspected cancer [25], and a retrospective survey in Kamuzu Central Hospital in Lilongwe, Malawi, reporting almost one-third of HIV-positive women having either high-grade dysplasia or cervical cancer [48]. Considering the 13% prevalence of HIV in Malawi and the 5% prevalence of cervical HPV 16 or 18 infections among women in the general population in Eastern Africa [14], a higher proportion VIA positive would be expected. Further research should be envisaged to better understand the real prevalence of VIA positivity and precancer lesions in Malawi.

Although only slightly more than 2% of women refused to participate in the survey, this represented every ninth women in Blantyre compared to less than 1% in Chiradzulu. MSF is very well-known in Chiradzulu District, as it has been supporting the health system in the district for many years, whereas MSF’s interventions in Blantyre only started with the cervical cancer screening project in 2018. The urban Blantyre population may have had less time and therefore been less willing to participate in the survey, especially as there are many surveys carried out in this city.

Although spatial sampling is an appropriate sample design for urban areas, cluster sampling would have been feasible for Blantyre as the city includes many non-residential areas, such as arable lands, fields, industrial areas, wasteland and nature reserves; and the population is fairly concentrated in certain areas. As well, the use of geospatial sampling in Blantyre City might have led to over-representation of wealthier women in the survey since they usually live in larger homes with a higher chance to be included in the survey, which may have biased the screening coverage results. However, the distribution of socio-demographic characteristics among participants is similar to the 2015-2016 Malawi Demographic and Health Survey [12], and the differences in socio-demographic characteristics between Blantyre and Chiradzulu rather more likely reflect the differences that usually exist between urban and rural areas than a sampling bias.

## Conclusions

Less than half of eligible women went for cervical cancer screening both in Blantyre City and in Chiradzulu District. These coverage results are slightly higher than previous surveys in Malawi, they are still much lower than the targeted cervical cancer screening rate of 80% for Malawi.

Most of the survey population had already heard about cervical cancer. Despite this knowledge, fewer than half of eligible women had been screened for cervical cancer. Reasons given for not attending screening are mutable concepts, such as lack of time, access, motivation and information, which are amenable to intervention. Most women who had been screened did so upon recommendation in a health facility, indicating not only that health care workers are successfully promoting screening to women already seeking care, and also that further efforts to inform and motivate women outside of the health facility on practical actions to prevent cervical cancer are necessary. Despite the low cervical cancer screening coverage, the positive reception amongst women who were screened, underscores this core part of cancer prevention as an important part of the way forward to reducing the burden of cervical cancer in Malawi. To significantly reduce mortality due to cervical cancer in Malawi requires a comprehensive health strategy that focuses on prevention, screening and treatment. Cervical cancer is not yet a disease of the past. Cervical cancer is a disease that can be prevented and treated; we know how, we now have to put it in place.

## Data Availability

The data sets generated during the survey are available from the corresponding author upon request.

## Abbreviations

ART: Anti-retroviral therapy
CC: Cervical Cancer
EAs: Enumeration Areas Chiradzulu District
HPV: Human papillomavirus
HIV: Human Immunodeficiency Virus
MoH: Malawi Ministry of Health
MSF: Médecins Sans Frontières
VIA: Visual inspection with acetic acid
